# Bacteriological and molecular characterization of *Mycobacterium bovis* isolates from tuberculous lesions collected among slaughtered cattle, Northwest Ethiopia

**DOI:** 10.1186/s12866-021-02349-1

**Published:** 2021-10-20

**Authors:** Mebrat Ejo, Belete Haile, Tsegaye Tariku, Seleshe Nigatu, Elias Kebede, Abebe Belete Bitew, Yitayew Demessie, Gashaw Getaneh, Atnaf Alebie, Musse Girma, Fusao Ota, Anwar Nuru

**Affiliations:** 1 Department of Biomedical Sciences, College of Veterinary Medicine and Animal Sciences, University of Gondar, P.O. Box 196, Gondar, Ethiopia; 2 Department of Veterinary Epidemiology and Public Health, College of Veterinary Medicine and Animal Sciences, University of Gondar, P.O. Box 196, Gondar, Ethiopia; 3 School of Veterinary Medicine, Woldia University, P.O. Box 400, Woldia, Ethiopia; 4 Department of Veterinary Pharmacy, College of Veterinary Medicine and Animal Sciences, University of Gondar, P.O. Box 196, Gondar, Ethiopia; 5 Aklilu Lema Institute of Pathobiology, Addis Ababa University, P.O. Box 9086, Addis Ababa, Ethiopia; 6 Medical Department, Seto Institute for Health Care, Tokushima University, 163-2, 7-chome, Dokihigashi, Kagawa-ken, Marugame-shi, 763-0082 Japan; 7 Department of Paraclinical Sciences, College of Veterinary Medicine and Animal Sciences, University of Gondar, P.O. Box 196, Gondar, Ethiopia

**Keywords:** Cattle, Genotypes, *Mycobacterium bovis*, RD4 deletion typing, Spoligotyping, Tuberculosis

## Abstract

**Background:**

In Ethiopia, the distribution of bovine tuberculosis (BTB) has long been known and documented as a major problem of animal health. However, the burden of circulating *M. bovis* strains is poorly understood in the country. Therefore; this study aimed to identify and characterize the mycobacterial isolates responsible for BTB in Northwest Ethiopia.

**Methods:**

A cross-sectional study was conducted on tuberculous lesions that had been collected from slaughtered cattle between September 2018 to June 2019. Collected lesions were cultured and tested for tuberculous bacilli. The MPT64 assay and Genotype line probe assay (LPA) were used for identification of mycobacterial isolates, and region of deletion 4 (RD4) typing and spoligotyping were used to characterize the *M. bovis* strains.

**Results:**

Of the total 1458 examined slaughtered cattle, only 62 (4.3, 95%CI; 0.0328–0.0542) had tuberculous lesions. The highest number of gross tuberculous lesions were observed from the lymph nodes of the thoracic cavity; at the mediastinal (40.3%, 25/62) and bronchial (22.6%, 14/62) lymph nodes. Of the 62 collected tuberculous lesions; 18 (29.0%) were culture positive for mycobacterium isolates, and only five isolates were confirmed for *M. tuberculosis* complex (MTBc) by the MPT64 assay and LPA. All the five MTBc isolates were positive for RD4 typing of *M. bovis* with a PCR product size of 446 bp, and no isolate was noticed to have *M. tuberculosis*. The detected *M. bovis* strains displayed five spoligotypes; with the common SB1176 and SB0133 *M. bovis* strains, although the two spoligotypes had not been previously reported.

**Conclusion:**

The present study shows that BTB in North Gondar, Ethiopia, is caused by *M. bovis* strains SB1176 and SB0033, with low frequency. Thus, the finding highlights the importance of continuous surveillance for mycobacterial strains in cattle populations.

**Supplementary Information:**

The online version contains supplementary material available at 10.1186/s12866-021-02349-1.

## Introduction

Bovine tuberculosis (BTB), caused by *Mycobacterium bovis* (*M. bovis*), is an infectious, chronic disease of cattle, which are known to be the primary hosts for this organism [1], although the disease has also been reported in different mammalian hosts, including humans [2]. The disease has high economic relevance within the context of livestock farming; directly affects animal productivity and also influences international trade of animal products [3, 4].

The World Organization for Animal Health (OIE) recognizes BTB as an important animal disease [1], and it presents enormous economic and public health problems in developing countries, including Ethiopia, mainly amongst the vulnerable area in rural populations [5]. The risk is supposed to be high in communities where close and frequent contact between humans and animals is common [5, 6]. According to reports [7], an estimated 68,900 new cases of zoonotic TB, due to *M. bovis*, occurred in Africa where pasteurization of milk is rare and BTB in cattle is common; however, *M. bovis* infection might be higher than the presently estimated.

In Ethiopia, the distribution of BTB in animal populations has long been described [8] and most studies also showed that BTB is prevalent mainly in cattle in wide areas of the country [9–11]. Absence of regular surveillance or any control measure strategies in the country aggravates the widespread and endemicity of BTB and being a threat to public health [12, 13]. In Ethiopia, different studies conducted so far on animals are based mostly on slaughterhouse reports made by researchers and veterinarians investigating gross tuberculous lesions of BTB, and also through the use of skin tuberculin testing [14], with a prevalence ranging from 4.7 to 10.2% for gross tuberculous lesions [10, 15, 16] and 9.7 to 50% when using tuberculin skin testing [13, 17]. Various studies have also described the importance of the slaughterhouse BTB surveillance in endemic situations; particularly, in countries with a low prevalence of BTB, between 5 and 15% of BTB infected herds were detected through the slaughterhouse surveillance [18–21]. However, these diagnostic tools cannot distinguish among members of the mycobacterial species, including *M. bovis*. A definitive examination can only be accomplished by isolation of *M. bovis* from clinical or post mortem samples [22, 23].

Several molecular methods, including RD4 deletion typing [24], spoligotyping [25], and other polymerase chain reaction (PCR)-based techniques [26], have been applied to genotype strains of the mycobacterial diseases and used to complement epidemiological studies on BTB. Although, in Ethiopia, few molecular studies have been used to genotype strains of the mycobacterial species and for molecular epidemiological studies on BTB [10, 27, 28]; there is still a lack of data on the strain diversity of mycobacterial species from slaughter cattle in the northwest of Amhara region. It was; therefore, aimed at molecular characterization of mycobacterial species (*M. bovis* strains) responsible for BTB in northwest Ethiopia.

## Materials and methods

### Study area

**Fig. 1:**
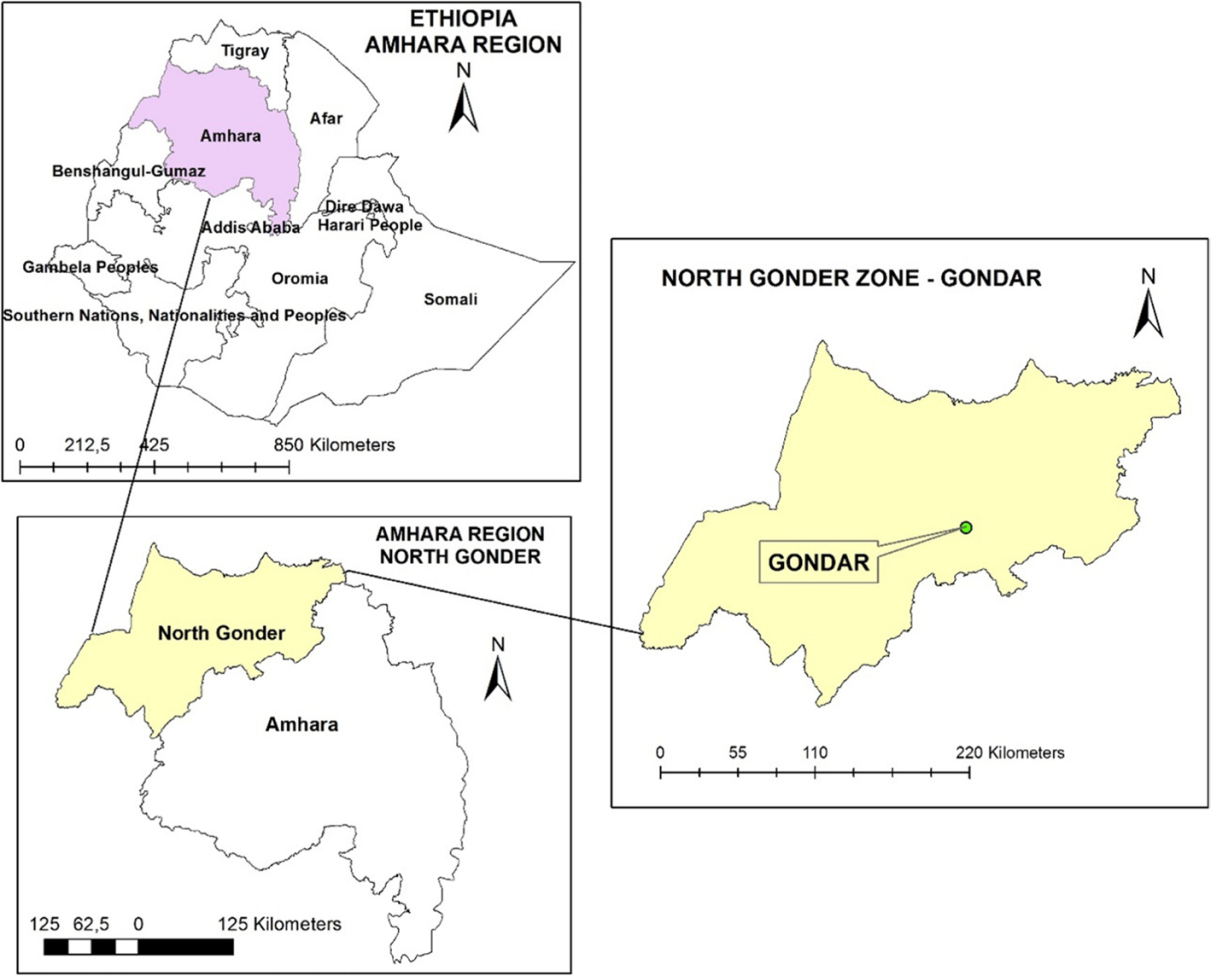
Map of the study area

The study was conducted at Gondar ELFORA abattoir, which is located 740 km away from the capital city, Addis Ababa, in the Northwest of Ethiopia, and located at the GPS coordinates of 12° 36′ 10.8648″ N and 37° 27′ 7.6752″ E, Fig. 1. The abattoir delivers animal slaughter services for Gondar town and the surrounding community; butcher houses, restaurants, hotels, and supermarkets. Cattle; brought to the abattoir for slaughter service, were purchased at various local markets in Gondar town and surrounding livestock rich districts, and transported to the abattoir. In this area, several people are involved in smallholder businesses, supplying food animals, and animal products to the communities and the slaughterhouses. According to Central Statistical Agency [29] report, North Gondar has an estimated 3.2 million cattle, 1.3 million sheep, and 1.8 goat population, and ranked first of the total 11 zones of the Amhara regional state in its livestock population.

### Study design and animals

A cross-sectional study design with a systematic random sampling method was performed between September 2018 to June 2019. For this study, all cattle that were presented for slaughter at the abattoir were enrolled as study animals. All of the cattle slaughtered at the abattoir were local zebu breed, and the sex of examined animals was male; while female animals were not slaughtered in an abattoir during this study. Subsequently, a total of 1458 cattle were examined for tuberculous lesions suspected of TB. Additionally, epidemiological data including breed, age, sex, and body condition score (BCS) of the individual animal were recorded during the study period. Body condition scoring of cattle was measured using a numerical scale ranging from 1 to 9 as described by Nicholson and Butterworth [30]; and BCS was categorized as poor (2 or 3 measurement scale), medium (4 to 6), and good (> 6).

### Antemortem and postmortem inspection

Cattle were examined physically before slaughter according to the antemortem inspection protocol [31]; particularly body conditions, condition of regional lymph nodes, and visible mucous membranes were examined for the individual animal.

Postmortem examination was carried out as described in the World Organization for Animal Health [32]. Briefly, lymph nodes of the head (retropharyngeal and mandibular), lymph nodes of the thoracic cavity (mediastinal and bronchial), and mesenteric lymph nodes were visualized, palpated, and incised into small size sections and then be inspected under bright light for the presence of tuberculous lesions. Organs including lungs, liver, kidneys, and intestines were also examined for visible lesions. Additional examinations of other lymph nodes and organs or body systems were considered whenever lesions are detected. If gross lesions suggestive of BTB are found in any of the examined tissues, the slaughtered animal was classified as tuberculous lesioned. Slaughter animals without any suggestive tubercular lesions were classified as non-lesioned [33].

### Sample collection and transportation

All tuberculous suspected tissue samples were collected in sterile screw-capped universal bottles with phosphate buffer saline (PBS) according to the World Health Organization (WHO) guidelines [34]. Following collection, the samples were transported by maintaining a cold chain to TB laboratory, University of Gondar Comprehensive Specialized Hospital, and kept stored at − 20 °C (a maximum of 3–4 weeks) until mycobacteriological analysis.

### Isolation of mycobacteria

Samples from suspected tuberculous lesions were processed and cultured following the protocol in World Organization for Animal Disease [32]. Briefly, the specimens were further sectioned into pieces using a sterile scalpel blade, and then ground and homogenized by pestle and mortar with the gradual addition of PBS. The homogenate was then transferred into a centrifuge tube and decontaminated by adding an equal volume of 4% NaOH, followed by centrifugation at 3000 rpm for 15 min. The supernatant was discarded, and the sediment was neutralized by 1% (0.1 N) HCl using phenol red as an indicator. Neutralization was achieved when the color of the solution changed from purple to yellow. Subsequently, 0.1 ml of the sediment from each sample was spread onto a slope of Lowenstein-Jensen (LJ) medium in duplicates; one enriched with sodium pyruvate while the other enriched with glycerol. Inoculated LJ media were incubated aerobically at 37 °C for about 8 weeks with weekly observation for mycobacterial growth.

### Identification of mycobacterial isolates

Preliminary identification of tubercle bacilli was done primarily with typical mycobacterial colony morphological characteristics on LJ media, and observation of acid-fast bacilli (AFB) using Ziehl–Neelsen (ZN) smear microscopy [34]. A rapid immunochromatographic MPT64 antigen test kit (SD Bioline, BD Diagnostic System, Sparks, MD) was used to identify mycobacterial species between *M. tuberculosis* complex (MTBc) isolates and nontuberculous mycobacteria (NTM). Strain identification was further confirmed by the Hain Genotype line probe assay (LPA) (Hain Lifescience GmbH, Nehren, Germany) [35] and Region of difference (RD4) deletion typing [24].

### DNA extraction

Deoxyribonucleic acid (DNA) extraction was done from culture-positive isolates by resuspending two loopful of bacteria in 200 μl 1 × TE buffer (10 mM Tris-HCl, pH 8, 1 mM EDTA), vortexed and boiled the mixture in a water bath for 5–10 min based on the template DNA isolation methods described elsewhere [24]. The extracted DNA suspension was stored at a − 20 °C freezer, and then later transported to Aklilu Lemma Institute of Pathobiology (ALIPB), Addis Ababa (Ethiopia) by keeping the cold chain in an icebox with an icepack for RD4-deletion typing and spoligotyping. Five μl of the DNA supernatant was used for PCR amplification.

### Region of difference (RD4) deletion typing

Region of difference (RD4) deletion typing was performed following the methods described previously [24] in a final volume of 20 μl for PCR amplification. In brief, the PCR products were electrophoresed on 1.5% agarose gel, and the product size was visualized using Syngene BioImaging System (Syoptics Group) for the presence or absence of RD4. Both *M. tuberculosis* H37Rv and *M. bovis* BCG DNAs were used as positive controls and distilled water as a negative control.

### Spoligotyping

Spoligotyping for MTBc strain typing was conducted following the protocol of the previous study [25]. Briefly, the amplified PCR products were hybridized to a set of 43 immobilized oligonucleotides at a spoligotype membrane for 60 min at 60 °C and followed by incubation (42 °C) in streptavidin-peroxidase conjugate for 45 min. Detection of hybridized DNA was performed by using chemiluminescent detection liquid and exposed to X-ray film. Both *M. tuberculosis* H37Rv and *M. bovis* BCG reference strains DNAs were included in each run as positive controls, and also distilled water as a negative control. The results were interpreted for the presence or absence of spacers from spoligotype images using the consensus of binary digits and an octal numbering system [25, 36].

### Genotype analysis

For genotype analysis, the publicly available spoligotype database (Mbovis.org) [37] was used to assign the *M. bovis* genotypes. An SB number was assigned when one or more isolates in the database shared the identical spoligotype-patterns, while spoligotype-patterns that had not been designated before were named as “New pattern or unknown”.

### Statistical analysis

Data analysis was performed using STATA® version 15.1 (StataCorp, USA). Descriptive statistics were used to analyze the overall proportion of tuberculous lesions and lesion frequency in different anatomical sites. Chi-square was used to evaluate the statistical significance of the associations of age and body conditions of cattle with the occurrence of tuberculous lesions. Binary logistic regressions were also used to investigate possible associations between the prevalence and the descriptive variables. A *p*-value of < 0.05 was considered statistically significant.

## Results

### Slaughter cattle characteristics and prevalence of BTB

A total of 1458 slaughtered cattle were examined in this study; all of which were male and local zebu breeds, with a higher proportion of the cattle (734, 50.3%) were grouped in the age category of 5–8 years old. The BCS showed 862 (59.1%) medium and 232 (15.9%) poor body conditioned, Table 1.

**Table 1:** Association between suspected tuberculous lesions and host related risk factors

Variable	No. of cattle examined (*n* = 1458) (%)	Positive for TB-lesion (*n* = 62) (%)	OR (95% CI)	*p*-value
Age category				
< 5 years	460 (31.6)	6 (9.7)	1.0	
5–8 years	734 (50.3)	39 (62.9)	4.23 (1.783–10.110)	0.001
> 8 years	264 (18.1)	17 (27.4)	5.21 (2.027–13.379)	0.001
Body condition score (BCS)				
Good	364 (24.0)	5 (8.1)	1.0	
Medium	862 (59.1)	50 (80.6)	4.42 (1.748–11.179)	0.002
Poor	232 (15.9)	7 (11.3)	2.23 (0.700–7.124)	0.174

*95%CI* Confidence Interval, *BCS* Body Condition Scoring, *OR* Odds Ratio

Of the total 1458 examined slaughtered heads of cattle, only 62 (4.3, 95%CI; 0.0328–0.0542) had suspected tuberculous lesions. Of these, 39 (62.9%) of the tuberculous lesions were detected in cattle age between 5 and 8 years old, Table 1. In the overall statistical analysis, suspected tuberculous lesions were most likely to be observed in cattle age category of 5–8 years old (OR = 4.23; 95%CI (1.783–10.110); *p* = 0.001), and in age greater than 8 years old (OR = 5.21; 95%CI (2.027–13.379); p = 0.001). Likewise, suspected tuberculous lesions were significantly higher in medium body conditioned cattle (OR = 4.42; 95%CI (1.748–11.179); *p* = 0.002), Table 1.

### Distribution of tuberculous lesions among tissues and organs

The highest number of suspected gross tuberculous lesions were observed from the lymph nodes of the thoracic cavity; particularly at the mediastinal (40.3%, 25/62) and bronchial (22.6%, 14/62) lymph nodes, and followed by the lung tissue (17.7%, 11/62). While the least distribution of tuberculous lesions was observed from lymph nodes of the head (retropharyngeal (5, 8.1%) and submandibular (5, 8.1%) lymph nodes), Table 2. The number of lesions per inspected cattle had only a single lesion in the examined anatomical sites of the slaughtered cattle.

**Table 2:** Distribution of BTB tuberculous suggestive lesions and bacterial growths in different lymph nodes and organs of slaughtered cattle

Sample source	distribution of BTB lesions	bacterial growth on LJ media with		total positive (%)
		Pyruvate, n (%)	Glycerol, n (%)	
mandibular	5	1 (20.0))	0 (0.0)	1 (20.0)
retropharyngeal	5	2 (40.0))	0 (0.0)	2 (40.0)
bronchial	14	3 (21.4)	1 (7.1)	4 (28.6)
mediastinal	25	5 (20.0)	2 (8.0)	7 (28.0)
lung	11	3 (27.3)	1 (9.1)	4 (36.4)
liver	2	0 (0.0)	0 (0.0)	0 (0.0)
total	62	14 (22.6)	4 (6.5)	18 (29.0)

### Isolation and identification of *Mycobacterium bovis*

In the present study, all 62 suspected tuberculous lesions were cultured on LJ media. Culture-positive isolates were identified in 29.0% (18/62) of the tuberculous suggestive lesions, Table 2, between 6 and 8 weeks of incubation with the majority on pyruvate enriched LJ media, 77.8% (14/18), and had flat, smooth, and non-pigmented colony characteristics. Forty samples (64.5%) had no growth in culture, and four samples were contaminated.

**Fig. 2:**
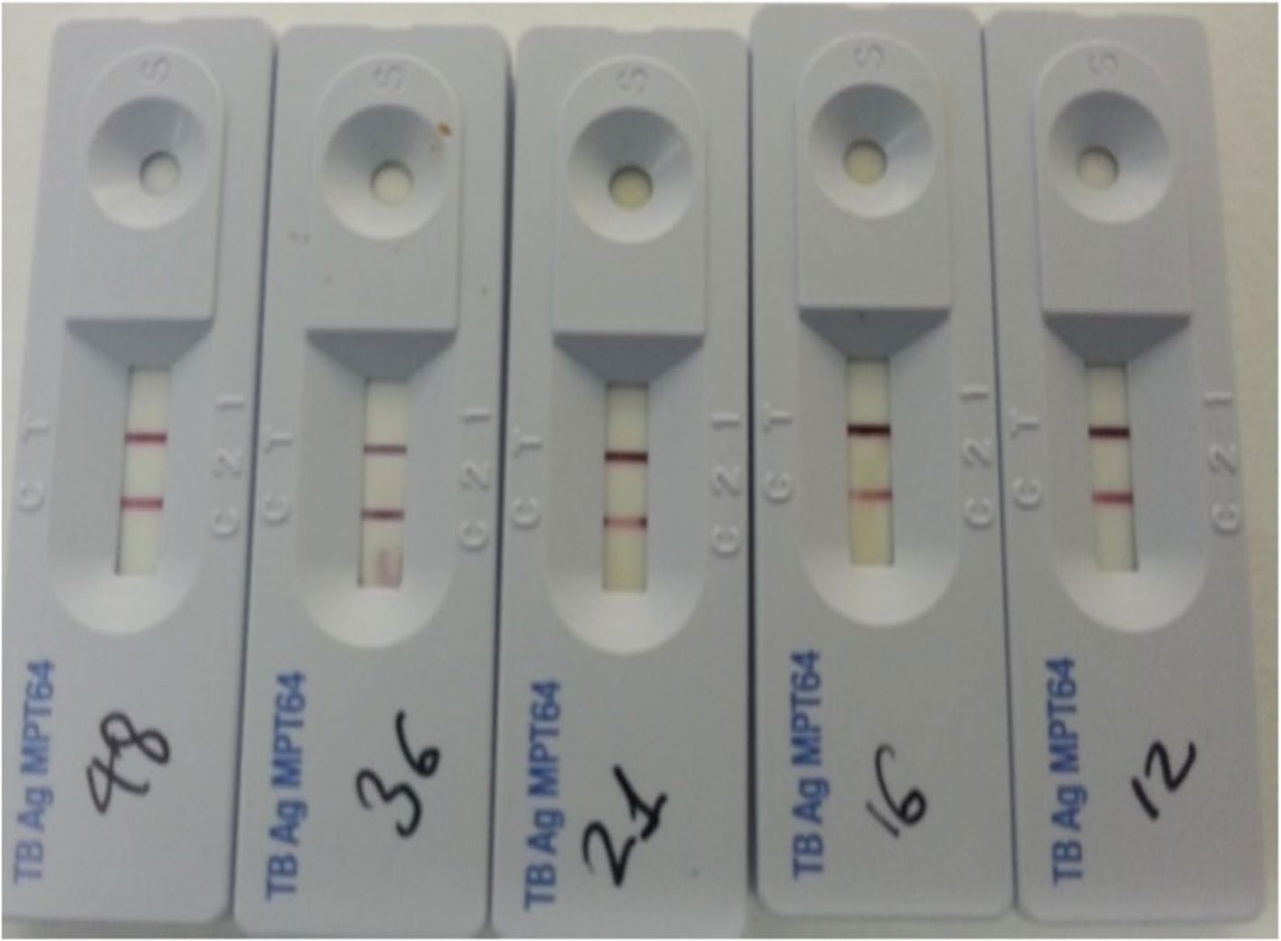
The MPT64 TBAg assay from five (27.8% of the total tested isolates) culture-positive isolates. The appearance of the pink band in the test region (T band) confirmed the presence of MPT64 antigen and identified as MTBc. The AgMPT64 test was done only once for each isolate

Among the 18 isolates, 10 (55.6%) were grouped as acid-fast bacilli (AFB) by smear microscopy (Ziehl–Neelsen) and colony characteristics; of which only five (27.8%) isolates were confirmed as members of the MTBc by the MPT64 TBAg test, Fig. 2, and Hain GenoType LPA, Table 3 and Supplement Fig. 1, while the remained 13 isolates were assumed to be non-tuberculosis mycobacteria (NTM).

**Table 3:** Mycobacterial strain identification using Hain Genotype line probe assay (LPA)

Isolate nr.	Line probe assay (LPA) result							Mycobacterial identification
	MTBc (TUB)	***rpo***B WT	***rpo***B MUT	***kat***G WT	***kat***G MUT	***inh***A WT	***inh***A MUT	
H37Rv	+	+	–	+	–	+	–	*M. tuberculosis*
Distilled water	–	–	–	–	–	–	–	None
07	–	–	–	–	–	–	–	non-MTBc
12	+	+	–	+	–	+	–	MTBc
13	–	–	–	–	–	–	–	non-MTBc
16	+	+	–	+	–	+	–	MTBc
20	–	–	–	–	–	–	–	non-MTBc
21	+	+	–	+	–	+	–	MTBc
23	–	–	–	–	–	–	–	non-MTBc
36	+	+	–	+	–	+	–	MTBc
37	–	–	–	–	–	–	–	non-MTBc
38	–	–	–	–	–	–	–	non-MTBc
41	–	–	–	–	–	–	–	non-MTBc
43	–	–	–	–	–	–	–	non-MTBc
45	–	–	–	–	–	–	–	non-MTBc
48	+	+	–	+	–	+	–	MTBc
50	–	–	–	–	–	–	–	non-MTBc
54	–	–	–	–	–	–	–	non-MTBc
60	–	–	–	–	–	–	–	non-MTBc
61	–	–	–	–	–	–	–	non-MTBc

The Hain Genotype LPA result from 18 culture-positive isolates obtained from LJ culture; five isolates (Isolate number: 12, 16, 21, 36, and 48) were confirmed as members of the *M. tuberculosis* complex (MTBc), while the remained 13 isolates were assumed to be non-tuberculosis mycobacteria (NTM)

*WT* Wildtype, *MUT* Mutation

### RD4 deletion typing

**Fig. 3:**
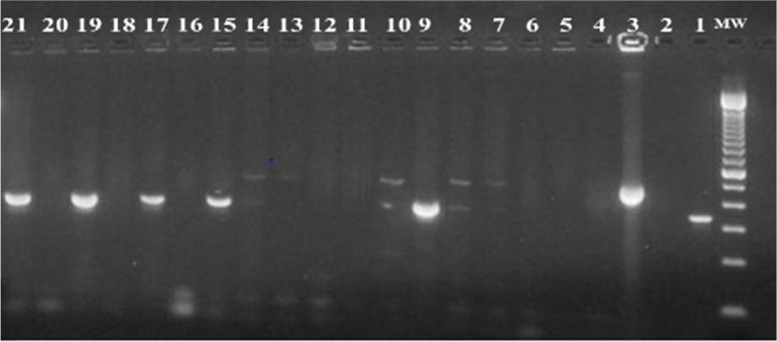
Gel electrophoresis of PCR products by RD4 typing of AFB and culture-positive isolates. (Lane 1: *M. tuberculosis* H_37_Rv DNA positive control; Lane 2: Distilled water as a negative control; Lane 3: *M. bovis* BCG DNA positive control; Lanes 4 to 21: PCR amplicons of culture-positive isolates DNAs; Lane MW: 100 bp molecular weight marker)

RD4 deletion typing of 18 isolates produced only five (27.8%) interpretable bands, Fig. 3. All the five isolates generated a PCR product of 446 bp, a characteristic of RD4 signal, and confirmed that they were *M. bovis* strains, while the remaining 13 (72.2%) isolates did not produce the band, and were identified as negative results.

### Spoligotyping of MTBc strains

Spoligotyping patterns were effectively detected in the five (27.8%) *M. bovis* strains among 18 isolates, while 13 (72.2%) were missed all spacers; of which five isolates were microscopy smear-positive. Besides, for the five MTBc strains with both MPT64 TBAg test, Hain Genotype LPA, and RD4 typing results available, spoligotyping also produced concordant MTBc strains in 100% (5/5).

Five spoligotypes of *M. bovis* were identified; three of them had been previously reported in the MBovis.com database, Table 4. The common spoligotypes were SB1176 (*n* = 2, 40%) and SB0133 (*n* = 1, 20%) of the *M. bovis* strains, while the remained two spoligotypes were not documented in the database “unknown patterns of the *M. bovis* strains”. One spoligotype (SB1176) was clustered and the other three strains were unique spoligotype patterns.

**Table 4:**
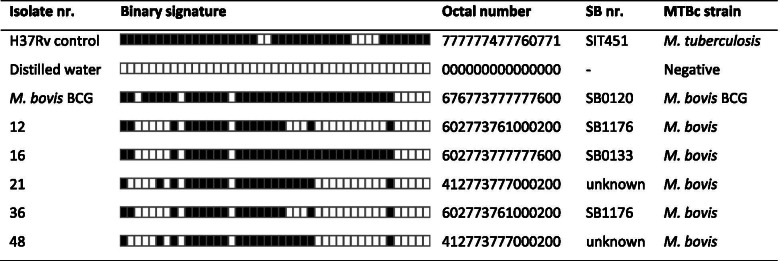
Spoligotype patterns of *M. bovis* isolates from slaughtered cattle in Northwest Ethiopia

We used *M. tuberculosis* H37Rv (absence of spacers 20–21 and 33–36) and *M. bovis* BCG (absence of spacers 3, 9, 16, and 39–43) as positive controls and distilled water as a negative control. The black squares indicated the presence of spacers and the white squares represent the absence of the spacers at positions 1 to 43 in the direct repeat locus

## Discussion

In the present study, we described the *M. bovis* strains among slaughtered cattle in northwest Ethiopia. The study showed that tuberculous lesions were detected in 4.3% of slaughtered cattle, which was consistent with other studies in Ethiopia [28, 38, 39], but lower than the reports elsewhere [15, 40]. Indeed, the prevalence of BTB varies from country to country, or from one place to another place within a country [14]. This variation might be linked to the type of animal production system [41, 42], and cattle breed [9, 43], while the extensive farming of zebu cattle breeds is commonly experienced in our study area that might not show the real picture of BTB prevalence. Studies have also supported that indigenous zebu cattle are relatively resistant to *M. bovis* infections, BTB [9, 41, 43, 44]. Our study animals were indigenous zebu cattle.

In our findings, culture-positive isolates were identified in 29% (18/62) of the tuberculous lesions, of which *M. bovis* was detected only in five isolates, all confirmed by MPT64 Ag assay and molecular tests, such as Hain Genotype LPA, RD4 typing, and spoligotyping. This agrees with the findings that explained *M. bovis* is isolated among slaughtered cattle in Ethiopia [10, 27, 28, 45]. However, our study displays a lower prevalence of *M. bovis* than other reports elsewhere [46–50]. In general, lower rates of *M. bovis* isolation from tuberculous lesions have been documented in the country [10], although the diagnosis of slaughter animals through abattoir-based inspection is the common practice in Ethiopia. Misdiagnosis of tuberculous lesions due to non-tuberculous mycobacteria and other granuloma-causing organisms might be the reason for a fewer number of *M. bovis* in our study; this is also in agreement with other studies [10, 51–53]. Moreover, the paucibacillary nature of calcified lesions, the influence of decontamination, and culture growth conditions in practice could also be the probable cause for the low efficiency of bacteriological isolation of *M. bovis* [23, 43, 51]. Nevertheless, our findings are consistent with the studies in other African countries [48–50], and hence revealed the distribution of *M. bovis* infection in cattle.

In this study, molecular analysis of the mycobacterial strains identified five spoligotypes in the Mbovis.org database [37], with the common SB1176 and SB0133 *M. bovis* genotypes. These *M. bovis* genotypes have also been reported as the most prevalent *M. bovis* strains in Ethiopia [10, 27, 28], and implicating the major cause of BTB circulating in Ethiopian cattle. Both *M. bovis* genotypes; SB1176 and SB0133, are members of the African 2 (Af2) clonal complex identified only in cattle adapted in East African countries, and characterized by the absence of spacers 3–7, 16, and 39–43 in their patterns [54]. The predominance of these *M. bovis* strains in cattle over this geographical area might certainly be explained by the uncontrolled free transboundary movement of the animal, probably with infected cattle or wild animals, within and between countries and neighboring areas [55, 56]. Also, the poor BTB control measures, including the absence of test and slaughter policy and insufficient quarantine measures might be the possible reasons for the spread and endemicity nature of the disease, BTB. Nevertheless, the two *M. bovis* spoligotypes (Octal number: 412773777000200) without assigned SB number (Mbovis.org) might therefore be emerged more recently and be distributed in our study area. It is, thus, important to note that further studies using highly polymorphic markers might need for a better knowledge of the circulating *M. bovis* strains in the region.

In the present study, no *M. tuberculosis* was isolated, however, various studies in Ethiopia have been reported both *M. bovis* as well as *M. tuberculosis* strains in cattle and humans [6, 10, 57, 58]; implicating the possible risk of *M. bovis* infection as zoonotic TB. In the country, due to the consumption habit of raw meat and unpasteurized milk, *M. bovis* should be considered to be a potential threat for human tuberculosis in a setting with a high prevalence of BTB and no control strategies.

This study had limitations. Because of the resource constraints and the limited investigation period, the study only included cattle slaughtered at the Gondar abattoir; the genotypes of *M. bovis* observed might not reflect the overall prevalence of these strains in the animal population of the region. In addition, we did not perform the statistical analysis for *M. bovis* spoligotypes distribution among slaughtered cattle, in which all cattle that were presented for slaughter were local zebu breed and male sex purchased from multiple markets in this study area.

## Conclusion

The current findings show that tuberculous lesions were common in cattle slaughtered in North Gondar, Ethiopia, and present the first insight of the strains of *M. bovis*, confirmed by the MPT64 antigen test, Hain Genotype LPA, RD4 typing, and spoligotyping. The study also reveals that *M. bovis* spoligotypes; SB1176 and SB0133, were the common mycobacterial strains identified from the tuberculous lesions collected among slaughtered cattle. Although the frequency is relatively low, the identified *M. bovis* strain suggests circulation of the pathogen in the cattle population of the study area and continues as a public health threat. Thus, the findings highlight the importance of continuous bacteriological and molecular investigations for mycobacterial strains in animal populations, and to assist the control strategies of BTB.

## Supplementary Information

**Additional file 1: Supplement Figure 1.** The Hain Genotype LPA result from 18 culture-positive isolates obtained from LJ culture.

## Data Availability

Data are available from the authors upon reasonable request.

## Abbreviations

AFB: Acid-fast bacilli
BCS: Body condition score
BTB: Bovine tuberculosis
LJ: Lowenstein-Jensen
LPA: Line probe assay
*M. bovis*: *Mycobacterium bovis*
MTBc: *Mycobacterium tuberculosis* complex
NTM: Nontuberculous mycobacteria
PCR: Polymerase chain reaction
RD4: Region of deletion 4
ZN: Ziehl–Neelsen
