# Supplementary material for: Bacteriological and molecular characterization of Mycobacterium bovis isolates from tuberculous lesions collected among slaughtered cattle, Northwest Ethiopia
Source: BMC Microbiol. 2021 Oct 20;21:286. doi: 10.1186/s12866-021-02349-1 (PMC8527785; doi:10.1186/s12866-021-02349-1)
Supplement: Supplementary file 1 — Additional file 1: Supplement Figure 1. The Hain Genotype LPA result from 18 culture-positive isolates obtained from LJ culture. [file 12866_2021_2349_MOESM1_ESM.docx]

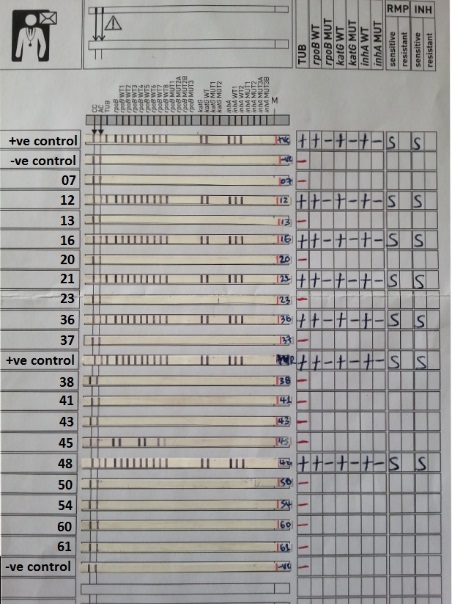


Supplement Figure 1. The Hain Genotype LPA result from 18 culture-positive isolates obtained from LJ culture.
